# Studies of Tumor Suppressor Genes via Chromosome Engineering

**DOI:** 10.3390/cancers8010004

**Published:** 2015-12-30

**Authors:** Hiroyuki Kugoh, Takahito Ohira, Mitsuo Oshimura

**Affiliations:** 1 Department of Biomedical Science, Institute of Regenerative Medicine and Biofunction, Graduate School of Medical Science, Tottori University, Yonago, Tottori 683-8503, Japan; ohira@med.tottori-u.ac.jp; 2 Chromosome Engineering Research Center (CERC), Tottori University, Yonago, Tottori 683-8503, Japan; oshimura@med.tottori-u.ac.jp

**Keywords:** telomerase, *TERT*, tumor suppressor gene, *PITX1*, melanoma, chromosome engineering

## Abstract

The development and progression of malignant tumors likely result from consecutive accumulation of genetic alterations, including dysfunctional tumor suppressor genes. However, the signaling mechanisms that underlie the development of tumors have not yet been completely elucidated. Discovery of novel tumor-related genes plays a crucial role in our understanding of the development and progression of malignant tumors. Chromosome engineering technology based on microcell-mediated chromosome transfer (MMCT) is an effective approach for identification of tumor suppressor genes. The studies have revealed at least five tumor suppression effects. The discovery of novel tumor suppressor genes provide greater understanding of the complex signaling pathways that underlie the development and progression of malignant tumors. These advances are being exploited to develop targeted drugs and new biological therapies for cancer.

## 1. Introduction

Cancer is the most common human genetic disease and is caused in part by alterations in DNA sequences and epigenetic regulation [1]. In particular, oncogenes and tumor suppressor genes, which are involved in activation and inhibition of cell proliferation, respectively, play a critical role in cancer development [2,3,4]. The concept of a tumor suppressor gene was originally developed with functional complementation, which inhibits transformed phenotypes through whole cell-cell fusion of normal and cancer cells; the two-hit theory that led to discovery of loss of heterozygosity (LOH) was also important. These studies demonstrated that compared to normal cells, tumor cells have recessive tumor-associated traits including tumorigenicity, *in vitro* transformed phenotypes, and immortality [5,6,7,8,9,10,11]. Additionally, hybrid cell clones frequently re-express tumor phenotypes (revertant) that are accompanied by the loss of specific chromosomes from normal cells [12,13]. These results suggest that putative tumor suppressor genes are localized on chromosomes that are lost from normal cells.

In 1971, Alfred Knudson proposed the two-hit theory in which accumulation of mutation(s) in a responsible gene on the two alleles inherited from each parent is required for cancer development [14]. LOH, resulting from deletion of one allele in a specific region, is thought to indicate the presence of a tumor suppressor gene [15,16]. In fact, the first tumor suppressor gene was identified by genome research studies of LOH in retinoblastoma. In 1985, Webster Cavenee concluded that homozygosity of a mutant allele on chromosome 13, the retinoblastoma (*RB*) tumor suppressor gene, is a likely prerequisite for development of retinoblastoma [17]. After intense research, the *Rb* tumor suppressor gene, which was identified as a key regulator of the cell cycle, was shown to be inactivated in various types of human tumors [18]. Thus, the presence of tumor suppressor genes was suggested by cell hybrid studies and LOH analysis [19,20,21,22,23,24,25].

Microcell-mediated chromosome transfer (MMCT), which is used to transfer a chromosome from normal somatic cells into human tumor cells, is also used as another approach for efficient identification of tumor suppressor genes [26,27]. We established a direct approach for identification of a chromosome carrying tumor suppressor gene(s) by introducing individual normal chromosomes into tumor cells [28,29,30,31,32,33,34,35,36,37,38,39,40,41]. In this review, we outline a general strategy for mapping the localization and identification of a functional tumor suppressor gene with MMCT.

## 2. Construction of Mouse A9 Clones Containing a Single Human Chromosome

**Figure 1:**
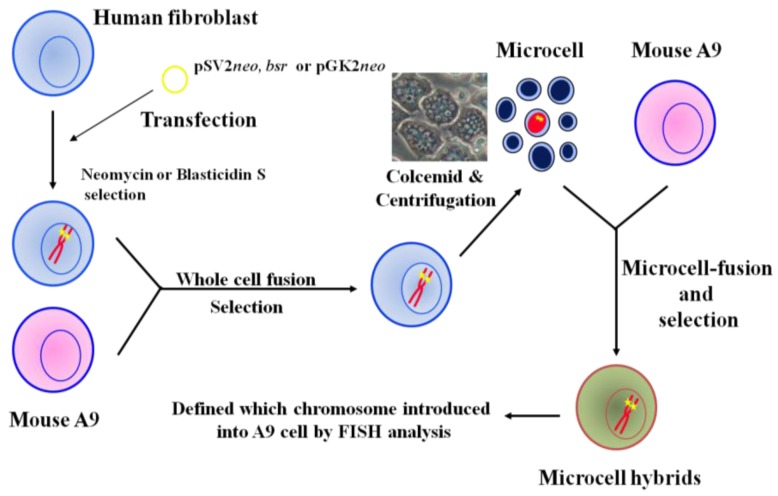
Construction of mouse A9 cells containing a single human chromosome.

Introduction of a normal chromosome into cancer cells via MMCT is an effective method for mapping and identifying tumor suppressor genes. To increase utilization of MMCT as a systematic approach for cancer research, we constructed a library of mouse A9 cells containing a single human chromosome, allowing any human chromosome to be introduced into target recipient cells such as cancer cells. The strategy we used for the mouse A9 monochromosomic library containing a single human chromosome is outlined in Figure 1. First, we transfected plasmids with pSV2*neo*, *bsr*, and pGK*neo* into normal human fibroblast cells, resulting in random integration of these dominant selectable markers into human chromosomes. Neomycin- and blasticidin S hydrochloride-resistant clones were isolated and fused to mouse A9 cells, producing many human/mouse hybrid clones that contain selectable marker-tagged human chromosomes. Finally, transfer of dominant selectable marker-tagged human chromosomes obtained from those hybrid cells into mouse A9 cells was performed with microcell fusion. To investigate the status of the introduced human chromosome, karyotyping and fluorescent in situ hybridization analysis of the isolated A9 microcell hybrid clones were performed. We have now generated A9 hybrids that each contain a human chromosome, except the Y chromosome [36,42,43].

## 3. The Use of MMCT to Identify Human Chromosomes that Carry Tumor Suppressor Genes

**Figure 2:**
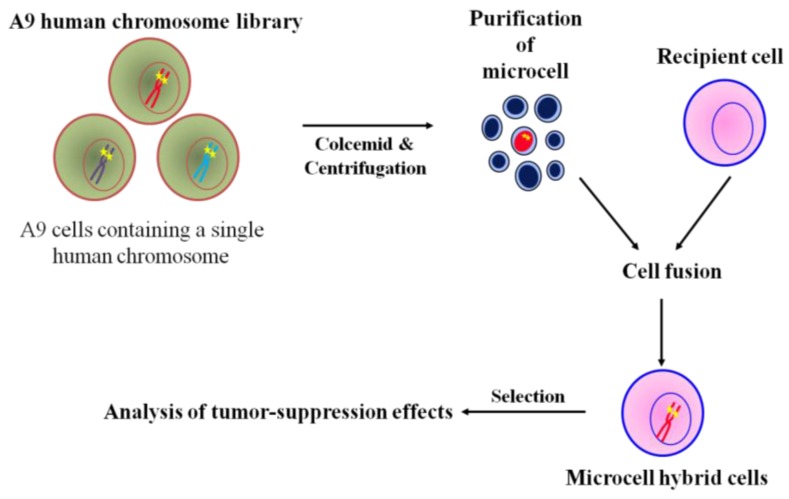
Chromosome transfer to cancer cells via MMCT.

The general microcell hybridization method is shown in Figure 2. Mouse A9 containing a single human chromosome with a dominant selection marker, *i.e.*, *neo* or *bsr*, were treated with colcemid for 48 h to induced micronuclei formation, which were then purified by cytochalasin B digestion and centrifugation. The isolated microcells were fused with recipient cell lines using polyethylen glycol. The cells were maintained in nonselective medium for 24 h, then trypsinized and split into three 90 mm dishes containing antibiotics. A specific human chromosome with a positive selectable marker into recipient cells was stably maintained under selective condition. Thus, MMCT is an invaluable approach in the functional study of tumor suppression effect of the genome [27]. Using the technique of MMCT, we and others showed that various human chromosomes contain many tumor suppressor genes that induce cellular senescence or metastasis (Table 1). For example, cellular senescence-related genes have been mapped to at least 10 different genetic loci, indicating restoration of the tumor suppression program in immortal cells [44,45,46,47,48,49,50,51]. Additionally, restoration of cellular senescence following introduction of chromosome 3, 6, 7, or 10 in human tumor cells is accompanied by repression of telomerase activity, which is involved in cellular immortalization [33,35,52,53]. Furthermore, we used MMCT to individually transfer each normal human chromosome, except for the Y chromosome, into mouse melanoma cells to clarify the functional role of tumor suppressor genes and to identify the human chromosome that carries these genes (Figure 2). We found that the effect of tumor suppressor genes could be classified into five types; Type 1: induction of cellular senescence; Type 2: suppression of *in vitro* growth properties and tumorigenicity; Type 3: suppression of tumorigenicity alone; Type 4: suppression of telomerase activity; and Type 5: inhibition of metastasis ability (Table 2) [36]. Thus, these findings from chromosome transfer research provide evidence that many tumor suppressor genes are implicated in the multistep process of development of malignant melanoma.

**Table 1:** The suppression effects of transformed phenotypes following introduction of normal human chromosomes into various tumor cell types.

Transferred Chromosome	Cell Lines	Type					Mapping Region	Reference
		1	2	3	4	5		
1	HT1080 (fibrosarcoma)		+	+				[30]
	TE85(osteosarcoma)	+						[44]
	143 B (TK-Ki-Ras-transformed TE85)	+						[44]
	CMV-Mj-HEL-1 (CMV-transfermed lung fibroblasts)	+						[44]
	10W-2 (immortal Syrian hamster fibroblasts)	+						[51]
	Isikawa (uterine endomerial carcinoma)	+						*
	HHUA (uterine endometrial carcinoma)	+	+					[45]
2	SiHa (cervical cancer)	+					2q37	[31,32]
3	RCC23 (renal cell carcinoma)	+			+		3p21.3	[40,46,54]
	KC12 (renal cell carcinoma)	+			+		3p14.2-p21.1	[33]
	HSC3 (oral squamous cell carcinoma)		+	+	+		3p21.2-p21.3 or 3p25	[41,55]
	TS1 (lung adenocarcinoma)	+						*
4	HeLa (cevical cancer)	+						[47]
	J82 (baladder cancer)	+						[47]
	T98G (glioblastoma)	+						[47]
5	A2058 (melanoma)		+		+			[26,39]
6	HALneo (immortal fibroblasts)	+						[48]
	LCS-AF.1-3 (immortal fibroblasts)	+					6p23-p24	[56]
	39neo (immortal fibroblasts)	+						[48]
	SV/HF (SV40-tranformed fibroblasts)	+						[48]
	HPV-16 (HPV-immortalized keratinocyte)				+			[53]
7	KMST-6 (immortal fibroblasts)	+						[49]
	SUSM-1 (immortal fibroblasts)	+						[49]
	CC1 (choriocarcinoma)	+						*
	R-3327 (rat prostatic cancer cells)					+	7q21-22, 7q31.2-32	[57]
	MeT5A (mesothelial cells)	+			+			[52]
8	R-3327 (rat prostatic cancer cells)					+	8p21-q12	[57]
10	Li7HM (hepatocellular carcinoma)	+			+		10p15.1	[35]
	R-3327 (rat prostatic cancer cells)					+	10q11-22	[57]
11	HeLa (cevical cancer)			+				[58]
	G401 (wilm’s tumor)		+	+				[59]
	SiHa (cervical cancer)			+				[29]
	A204 (rhadboyomyosarcoma)			+				[29]
	HHUA (uterine endometrial carcinoma)			+				[29]
	HT1080 (fibrosarcoma)			+				[30]
	RD (rhabdomyosarcoma)	+						[50]
	H15 (bladder cancer)	+						*
	R-3327 (rat prostatic cancer cells)					+	11p13-11.2	[57]
13	R-3327 (rat prostatic cancer cells)					+		[60]
17	R-3327 (rat prostatic cancer cells)					+	12p11-q13, 12q24-ter	[57]
18	HHUA (uterine endometrial carcinoma)	+	+					[45]
X	HocB (ovarian carcinoma)	+						*
	ELCO (breast carcinoma)	+						*

Type 1: Induced senescence; Type 2: Suppression of *in vitro* transformed; Type 3: Suppression of tumorigenicity; Type 4: Suppression of telomerase activity; *: Unpublished data by the authors; +: Effective.

**Table 2:** The suppression effects of transformed phenotypes following introduction of normal human chromosomes into the mouse melanoma B16-F10 cell line.

	Type 1	Type 2	Type 3	Type 4	Reference
**Transferred Chromosome**	1, 2	5, 7, 9, 10, 11, 15, 16, 19, 20 or 22	5, 7, 9, 10, 11, 13, 14, 15, 16, 19, 20, 22 or X	5	[36,37,38]

Type 1: Induced senescence; Type 2: Suppression of *in vitro* transformed; Type 3: Suppression of tumorigenicity; Type 4: Suppression of telomerase activity.

## 4. Mapping of Tumor Suppressors on Human Chromosomes

To identify the location of the tumor suppressor genes in specific chromosome regions, we utilized three tools: revertant clones, sub-chromosomal transferrable fragments (STFs), and truncated chromosomes.

### 4.1. Deletion Mapping Using Revertant Clones

**Figure 3:**
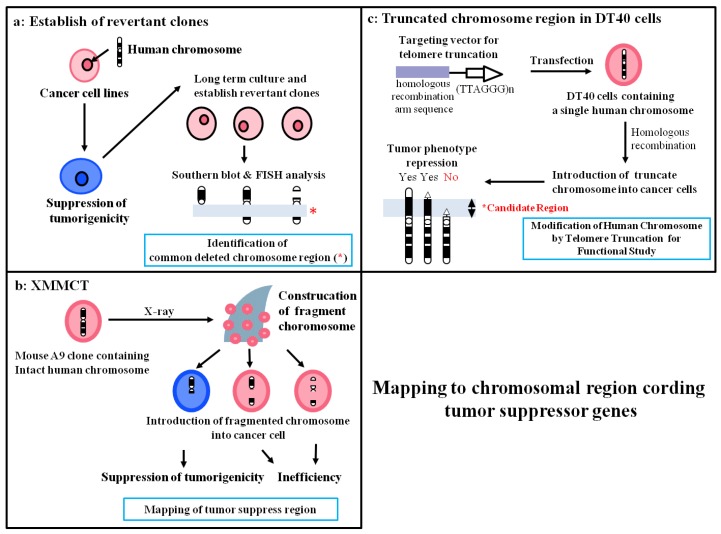
Mapping of chromosome regions which coding tumor suppressor genes via the modification of MMCT.

Introduction of many human chromosomes can suppress the transformed phenotype of various cancers, suggesting that several tumor suppressor genes are present on various chromosomes and play a crucial role in the development or progression of cancer. On the other hand, the appearance of revertant microcell hybrid cells that have escaped from suppression effects occurs at a fixed frequency in late-passages cultures, resulting in loss of tumor suppressor genes or regions on the introduced chromosome (Figure 3a). Therefore, investigation of common deleted regions in revertant microcell hybrid clones is a valuable research method for mapping tumor suppressor genes (Table 1) [33,35].

### 4.2. Construction of STFs by X-Irradiation Chromosome Transfer (XMMCT)

Occurrence of spontaneous loss in regions that carry tumor suppressor genes is believed to be required for acquisition of revertant microcell hybrid clones. Therefore, to improve the efficiency of mapping the localization of putative tumor suppressor genes by functional analysis, we generated various STFs using X-rays (Figure 3b). We previously identified the presence of senescence-related gene(s) in a 450- to 600-kb region on human chromosome 1q42.3 using a combination of functional analysis using STFs in the mouse melanoma cell line B16-F10 and deletion mapping of revertant clones that escaped from cellular senescence [38]. Moreover, using the same approach, we reported mapping of a novel cellular senescence-related gene on human chromosome 2q37 [32]. Thus, we provided evidence that functional STFs are useful for identification of the region on the chromosome that contains a tumor suppressor gene.

### 4.3. Construction of Truncated Chromosomes by Chromosome Engineering

Telomerase is activated in most cancer cells and plays an essential role in the immortality of cancer cells via elongation of telomere length. Human telomerase reverse transcriptase (*hTERT*) is the catalytic protein component of telomerase that synthesizes telomeric DNA directly onto the ends of chromosomes by reverse transcription of the RNA template [61,62,63,64]. Because the maintenance of telomere stability is almost universally required for the long-term proliferation of tumor cells, cells escape from cellular senescence and become immortal by activating telomerase. On the other hand, telomerase activity is precisely regulated in normal human somatic cells, suggesting that the telomerase control system may have originated as an antitumor defense mechanism. Using the MMCT method, we and others have shown that human chromosomes 3, 5, 6, 7, and 10 may carry *hTERT* suppressor genes [33,35,37,39,41,52,53]. Here, we focus on our description of mapping of an *hTERT* repressor on chromosome 3 via the chromosome engineering technique. We reported that introduction of human chromosome 3 using MMCT represses *hTERT* transcription in human renal carcinoma RCC23 cells with telomerase activity, resulting in induction of cellular senescence. To identify the precise location of the *hTERT* repressor gene on this chromosome, we developed a unique experimental approach involving functional analysis of various truncated chromosome 3 fragments that are produced by the chromosomal engineering technique (Figure 3c). We used chicken DT40 pre-B-cells, which a have high ability for homologous recombination, to produce truncated chromosome 3 [65]. First, a neomycin-tagged intact human chromosome 3 (#3) was transferred into DT40 cells (DT40#3) by MMCT. Second, we generated three targeting vectors using different homologous sequences: 1) 10 kb from the 3p24 locus (for deletion from 3p24-telomere; #3delp24-pter), 2) ~4 kb from the 3p22 locus (for deletion from 3p22-telomere; #3delp22-pter), and 3) ~8 kb from the 3p21.3 locus (for deletion from 3p21.3-telomere; #3delp21.3-pter). Third, we transfected these targeting vectors into DT40#3 cells and looked for generation of truncated chromosome 3. To confirm whether accurate recombination had occurred with the targeting vector on chromosome 3 in DT40#3 cells, we performed PCR and southern blotting analysis using Sequence-Tagged-Sites markers located on human chromosome 3. We transferred each truncated chromosome 3 into mouse A9 cells to increase the efficiency of chromosome transfer into RCC23 cells. Finally, we isolated three truncated chromosomes: #3delp24-pter, #3delp22-pter, and #3delp21.3-pter. To determine the region that contains *hTERT* suppressor gene(s) on human chromosome 3, these truncated chromosomes were transferred into RCC23 cells. Microcell hybrid clones generated by introduction of truncated human chromosome 3 (3pdelp24-pter and #3pdelp22-pter) completely inhibited *hTERT* transcription. On the other hand, the remaining #3delp21.3-pter had no effect on *hTERT* transcription. These results indicated that the *hTERT* suppressor gene(s) are localized in the 3p21.3-p22 region that was commonly retained in the truncated chromosomes (#3delp24-pter and #3 delp22-pter, but not #3pdel21.3-pter). PCR analysis with several Sequence-Tagged-Sites markers was used to narrow down the 3p21.3-p22 region that carries the *hTERT* suppressor gene(s). A 7-Mbp interval between D3S3597 and D3S1573 in the 3p21.3 region was found to be necessary for suppression of *hTERT*, suggesting that this minimal region controls telomerase activity by suppressing *hTERT* expression in RCC23 cells [40]. These results provided evidence that functional analysis by production of truncated chromosomes in DT40 cells is a potentially valuable and unique approach for identifying regulatory factors that are responsible for the suppression of *hTERT* transcription.

## 5. Identification of a Functional Suppressor Gene with a Combination of MMCT and Microarray

**Figure 4:**
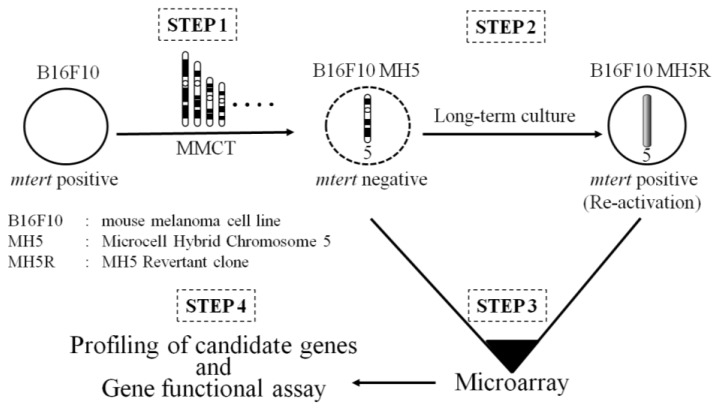
Identification of a functional suppressor gene by combination of MMCT and microarray.

Using MMCT, we individually transferred each normal human chromosome, except for the Y chromosome, into the mouse melanoma cell line B16-F10. Introduction of chromosomes 1 and 2 induced cellular senescence. Microcell hybrid clones that contain chromosomes 5, 7, 9, 10, 11, 13, 14, 15, 16, 19, 20, 21, 22, and X significantly inhibited cell proliferation. These results indicated the presence of at least two cellular senescence-related genes and many putative tumor suppressor genes in B16-F10 cells that are implicated in the multistep process of neoplastic development. In particular, compared to parental B16-F10 cells, B16-F10 microcell hybrid clones with human chromosome 5 (B16-F10 MH5) strongly suppressed both tumorigenicity and *in vitro* growth properties including serum-independent growth, cell proliferation, saturation density, and colony-forming efficiency in soft agar [36]. Furthermore, this phenomenon was accompanied by inhibition of telomerase activity and mouse *tert* (*mtert*) transcription [37]. Thus, human chromosome 5 may carry gene(s) that can negatively regulate *mtert* transcription. The expression of *mtert* was inhibited in B16-F10 MH5 clones, but not late-passage MH5 clones (MH5R), suggesting that MH5R clones may result in loss of function of a candidate *mtert* suppressor gene on the introduced chromosome 5. To identify the *mtert* suppressor gene(s) on human chromosome 5, we observed a change in the expression profile with the introduced human chromosome 5, except for the signals derived from mouse, between MH5 and MH5R clones. As a result, we identified paired-like homeodomain 1 (*PITX1*), which is more highly expressed in the MH5 clones than MH5R (Figure 4). *PITX1*, which is located on human chromosome 5, was originally described as a member of the bicoid class of homeodomain proteins. The transcription factor is recruited to regulate the transcription of pro-opiomelanocortin (*POMC*) in the adult pituitary [66]. *PITX1* also regulates the developing hindlimb, but not the forelimb, in mice [67,68]. Additionally, *PITX1* acts as a negative regulator of the *RAS* pathway through *RAS* protein activator-like 1 (*RASAL1*), a member of the *RAS* GTPase-activating protein family 5 [69]. *PITX1* also induces activation of *p53* in human breast cancer cells by directly binding to its promoter region [70]. Downregulation of *PITX1* was reported in various types of human cancer including colon, prostate, bladder, lung, and gastric cancers, Barrett’s adenocarcinoma, oral tumors, and malignant melanoma [69,71,72,73,74,75]. This evidence suggests that *PITX1* plays a crucial role in cancer development. Overexpression of *PITX1* in B16-F10 cells and the human melanoma cell line A2058 induces downregulation of *mtert* and *hTERT* transcription, and knockdown of *PITX1* with siRNA in B16-F10 MH5 and A2058 MH5 leads to increased *mtert* and *hTERT* expression. Additionally, co-transfection of an *hTERT* and *mtert* promoter construct with *PITX1* in human and mouse melanoma cells inhibits the activities of both promoters. Furthermore, we found that three and one binding sites within a unique conserved region of the *hTERT* and *mtert* promoters, respectively, are responsible for transcriptional activation of *hTERT* and *mtert*. Thus, *PITX1* is a negative regulator of *TERT* through direct binding to the *TERT* promoter, which ultimately regulates telomerase activity. These data suggest that *PITX1* on human chromosome 5 is a negative regulator of *TERT*, strongly suggesting that a combination of MMCT and gene expression profile analysis is a powerful approach for identification of a functional tumor suppressor gene [26]. Furthermore, we recently reported that microRNA-19b (miR-19b) regulates *hTERT* expression and cell proliferation through inhibition of *PITX1* [76]. Thus, identification of a novel tumor suppressor gene will lead to increased understanding of the molecular mechanisms of cancer development.

## 6. Conclusions

In this review, we describe mapping to identify tumor suppress genes by various functional analysis methods that are based on the chromosome engineering technique (MMCT). A large number of tumor suppressor genes have also mapped to specific chromosome regions by restriction-fragment-length-polymorphism (RFLP) and comparative genomic hybridization (CGH) analyses of various human tumors [15,25]. However, RFLP and LOH are not always detected in normal and tumor cells, respectively. The altered phenotypes exhibited by the introduction of a specific chromosome *via* MMCT have allowed to identify chromosomes containing a specific cellular function of the tumor suppressor genes without LOH. However, a weak point is the low efficiency of microcell fusion containing a target chromosome. The development of improved technologies for increasing the MMCT frequency will facilitate the isolation of tumor suppressor genes [77,78].

Although we identified a tumor suppressor gene as a novel negative regulator of telomerase with a combination of chromosome engineering and expression profiling analysis, this strategy is more difficult for detecting non-coding RNAs including miRNAs and long non-coding RNAs. Recent studies showed that both types of RNAs play an important role in cancer development [79,80,81,82,83,84]. Therefore, the combination of chromosome engineering and high-throughput RNA sequencing will be a useful functional analysis approach for identification of novel non-coding tumor suppressor genes. Further studies involving detailed investigation of transcription analysis in tumor cell clones by introduction of a normal human chromosome will contribute greatly to novel anti-cancer gene discovery and increased understanding of the complex pathways in cancer development. These advances are being exploited to develop targeted drugs and new biological therapies for cancer.
